# Characterization of herbicide-resistant *Eucalyptus* plants expressing phosphinothricin acetyltransferase gene

**DOI:** 10.1186/1753-6561-5-S7-P135

**Published:** 2011-09-13

**Authors:** Esteban González, Carla Gugliermoni, Milton Galvão, Maria Fagundes, Maria Ferreira, Guilherme Almeida, Henrique Alves, Jose Gonsalves, Fernando Silva, Sergio Bentivenha, Shinitiro Oda, Eduardo Mello

**Affiliations:** 1 FuturaGene Brazil Ltda, Brazil; 2 Suzano Papel e Celulose, Brazil

## 

Herbicide resistant crops are commercially advantageous for efficient field productivity by enabling use of non-selective herbicides for weed management. Herbicide resistant trees could be used to improve productivity and reduce the costs of forest management through to the first and second years post tree establishment. We describe the introduction of the *phosphinothricin acetyltransferase* (*bar*) gene, which confers resistance to the herbicide glufosinate, into *Eucalyptus pellita* (clone EP11) and hybrid *Eucalyptus* (*E. grandis x E. dunni* - clone SP1383) under the control of a constitutive promoter. The transgenic lines produced showed a copy number ranging from 1 to 3 copies by Southern blot analysis. Herbicide resistance of the transgenic clones was assessed in the greenhouse by application of 200g/L phosphinotricin at the level of 6.0 L/ha and 4.0 L/ha (commercial rate). The effect of the two herbicide treatments was tested on 4-week and 6-month old plants derived from several transgenic events from the two different *Eucalyptus* clones. All 4-week old non-transgenic control plants, as well as whole shoots of 6-month old control plants were killed by the herbicide by fifteen days after application. The same herbicide treatment effectively killed all the weeds in field conditions. All the transgenic plants showed strong resistance to the herbicide in all treatments with no observed negative effects. The use of herbicide resistant trees may provide an additional means for improving the economic efficiency of plantation forest management.

